# Ultrasound-guided unilateral versus bilateral erector spinae plane block for postoperative analgesia of patients undergoing laparoscopic cholecystectomy

**DOI:** 10.1515/med-2025-1268

**Published:** 2025-10-13

**Authors:** Ayça Tuba Dumanlı Özcan, Yusuf Yılmaz, Mustafa Turan, Erdal Özcan, Ezgi Erkılıç, Handan Güleç

**Affiliations:** Bilkent City Hospital, University of Health Sciences, Ankara, Türkiye; Anesthesiology and Reanimation, Ankara Bilkent City Hospital, Ankara, Türkiye; Anesthesiology and Reanimation Department, Ankara Yıldırım Beyazıt University, Ankara, Türkiye

**Keywords:** ESPB, erector spinae plane block, tramadol, bupivacaine, cholecystectomy, laparoscopic, pain, regional anesthesia

## Abstract

**Introduction:**

Ultrasound-guided erector spinae plane block (ESPB) was originally developed for the treatment of neuropathic chest pain and has since been used in various thoracic, lumbar, and sacral surgeries.

**Objective:**

This study aimed to establish whether unilateral or bilateral ESPB is more effective for pain management in laparoscopic cholecystectomy.

**Materials and methods:**

A total of 54 adult patients undergoing laparoscopic cholecystectomy were divided into three groups: unilateral ESPB, bilateral ESPB, and a control group (no ESPB). The unilateral ESPB group received 20 mL of 0.25% bupivacaine preoperatively at the T8 vertebral level. The bilateral ESPB group received 20 mL of 0.25% bupivacaine to both sides of the vertebra. The control group received no intervention, and all three groups received general anesthesia. Intraoperatively, all patients received 50 mg of dexketoprofen and 1 mg/kg of tramadol. Postoperative tramadol use and visual analog scale (VAS) scores were recorded at 0 min, 30 min, 2 h, 6 h, 12 h, and 24 h.

**Results:**

Demographic characteristics did not differ significantly between the groups. At the 6 h post-surgery, the VAS scores in the bilateral ESPB group were significantly lower than the control group (*p* < 0.001). Total tramadol use in 24 h was significantly lower in the bilateral ESPB group compared with the control group (*p* = 0.003).

**Conclusions:**

Bilateral ESPB could be a valuable component of multimodal analgesia strategies in laparoscopic cholecystectomies.

## 1 Introduction

Forero et al. developed an ultrasound-guided erector spinae plane block (ESPB) for the treatment of neuropathic chest pain, which has since been used in various thoracic, lumbar, and sacral surgeries [1]. ESPB is a regional anesthetic technique that involves the ultrasound-guided injection of local anesthetic into the paraspinal region, aimed at reducing both visceral and somatic pain. Pain following laparoscopic cholecystectomy is associated with both trocar entry incisions in the abdominal wall and distention caused by pneumoperitoneum, along with somatic pain originating from the T6–L2 nerves. Laparoscopic cholecystectomy is a routine surgical procedure in adults and can result in various types of pain, including visceral (e.g., intra-abdominal pain), incisional (e.g., somatic pain), and referred somatic pain (e.g., shoulder pain), due to factors, such as tissue damage, diaphragmatic irritation, and residual pneumoperitoneum [2]. Therefore, analgesic options that effectively address all sources of pain should be prioritized [3]. ESPB provides both somatic and visceral analgesia and can be beneficial for this procedure [4].

There have been cases in the literature where unexpected bilateral effects were observed, even when unilateral ESPB was performed. A cadaveric study concluded that the reason for bilateral ESPB was increased pressure from insufflation, which affected the spread of the local anesthetic [5]. Additionally, the local anesthetic could have dispersed depending on the patient’s position due to gravitational effects. Previous studies have shown that the spread of local anesthetic is influenced by the patient’s position during an epidural block [6]. Unilateral ESPB can offer advantages, such as shorter procedure time, reduced local anesthetic use, fewer risks of complications, and effective analgesia with a single injection. As such, the aim of this study was to compare unilateral and bilateral blocks to establish whether unilateral ESPB is sufficient for this type of surgery.

## 2 Materials and methods

### 2.1 Study design

For this prospective randomized study, the adult case, who underwent laparoscopic cholecystectomy surgery in the American Society of Anesthesiologists (ASA) I–II group under general anesthesia, was informed and included in the study after written consent was given. The inclusion criteria were that the participants were aged 18–65 years, had ASA status I–II, and had a body mass index (BMI) of 23–35. Those with an ASA physical status III and higher; renal failure requiring hemodialysis; liver cirrhosis leading to liver dysfunction (e.g., prolonged international normalized ratio and increased basal bilirubin); known dementia, stroke, or other central nerve system disease; a history of severe psychiatric illness, alcohol consumption, or patients with drug abuse; multiple trauma and head trauma; and patients with a BMI of greater than 35 were excluded from the study.

### 2.2 Material and methods

A total of 54 patients were randomly assigned to either group 1 (control, *n* = 17), group 2 (unilateral ESPB, *n* = 20), or group 3 (bilateral ESPB, *n* = 17) (Figure 2). Random assignment of the patients was performed using the closed envelope method. The patients were taken to the operating room and a non-invasive blood pressure cuff, pulse oximetry probe for peripheral oxygen saturation (SpO_2_), and precordial ECG electrode in the standard D2 lead were placed. Vascular access was established with an 18–20 G intravenous cannula on the back of the hand, and the calculated maintenance was given as a crystalloid. After preoxygenation with 100% oxygen for 3 min, induction was performed with lidocaine (1–1.5 mg/kg), propofol (2–3 mg/kg), remifentanil (1 µg/kg), and rocuronium (0.6–1.2 mg/kg) were administered intravenously. After waiting for sufficient time, the patients were intubated endotracheally. Basal ventilation tidal volume was set to 6–8 mL/kg, frequency to 12, and fraction of inspired oxygen (FiO_2_) to 50%, but then the ETCO_2_ was kept at 30–36, and changes were made so that the peak pressure remained below 38 cm H_2_O. For maintenance, a 50:50 ratio of O_2_ to air was maintained, and 3% sevoflurane was administered. All patients received 50 mg dexketoprofen intraoperatively. The patients’ systolic arterial pressure (SAB), diastolic arterial pressure, mean arterial pressure, peak heart rate, oxygen saturation (SO_2_), and end-tidal CO_2_ (ETCO_2_) values were followed throughout the operation and recorded at 5 min intervals. The group I patients (control) were not treated with ESPB. For group 2, a unilateral block was performed with 0.25% 20 mL bupivacaine under ultrasound guidance, and group 3 received a bilateral ESP block. The ESP block was applied immediately before the induction of general anesthesia. ESP was performed after skin preparation and under ultrasonographic guidance using a linear 3–13 MHz ultrasound probe (SonoSite S-Nerve, Bothell, WA98021, USA). The ultrasonographic probe was placed longitudinally and parasagittally 3 cm lateral to the T8 spinous process of the thoracic vertebra. The T8 transverse process of the erector spinae muscles was defined as superficial. A 22-G 8 cm needle (sonoPlex STIM, PAJUNK, Germany) was used.

The tip of the needle was inserted into the plane of the fascia from the deep face of the erector spina muscle, and its position was confirmed by ultrasonography of the elevating view of the erector spina muscle of the needle tip and of fluid dissemination in the bony body of the transverse process. In total, 20 mL of 0.25% bupivacaine was injected on one side of the T8 spinous process for group 2 and both sides for group 3. No additional local anesthetic was applied to the trocar entry sites.

Tramadol dose as postoperative rescue analgesia and the visual analog scale (VAS) values at 0 min, 30 min, 2 h, 6 h, 12 h, and 24 h were recorded. T0 is defined as the first postoperative minute immediately after extubation at the end of surgery. Pain intensity was compared between groups using a VAS. VAS is a subjective scale that is graded 0–10 and was used to measure the level of pain. The anesthesiologist who randomly assigned the patients also performed the blocks for all participants but was not involved in the collection of postoperative data or its analyses.

### 2.3 Outcome measures

The primary outcome measures of the study were the VAS pain scores that were recorded at 0 min, 30 min, 2 h, 6 h, 12 h, and 24 h. The secondary outcomes were additional rescue analgesic requirement and total amount of tramadol used in 24 h. Rescue analgesia (intravenous tramadol 50 mg) was given if the pain score was 4 or higher on VAS during 1–24 h. If the pain remained the same in the following 1-h period, further intravenous tramadol (50 mg) was given. Furthermore, shoulder pain, nausea, vomiting during the first 24 h, and duration of discharge were noted.

### 2.4 Sample size

Based on a prospective, single-blind, randomized clinical study (J Clin Anesth. 2018; 50:65-68. doi: 10.1016/j.jclinane.2018.06.033) evaluating the effectiveness of the ESPB for postoperative analgesia management in laparoscopic cholecystectomy, the effect size calculated with the mean and standard deviation values of tramadol use in the first 24 h after surgery (effect size *f* = 0.48), alpha error (*p* value) was 0.05, and 1-beta error (power) was 0.80, assuming that the null hypothesis is tested. A total of 45 people (15 per group) would be enough to complete the study. G Power Statistical Program version 3.1.9.4 (Universität Düsseldorf, Germany) was used for analyses.

### 2.5 Statistical analyses

Data analyses were performed with IBM SPSS Statistics 25.0 software (IBM Corporation, Armonk, NY, USA). The near-normality of the distribution of continuous numerical variables was examined using the Shapiro–Wilk test. The Levene test was used to test the homogeneity of variances. Descriptive statistics were expressed as mean ± standard deviation or median (25th percentile–75th percentile) for continuous numerical variables and as number of cases (%) and for categorical variables. The significance of differences between groups with respect to the mean was assessed using one-way analysis of variance (ANOVA), whereas the significance of differences with respect to continuous numeric and sortable variables where the assumptions of the parametric test statistic were not met were examined by the Kruskal–Wallis test. If the Kruskal–Wallis test results were significant, Dunn–Bonferroni multiple comparison tests were used to identify the group(s) causing the said difference. Categorical variables were assessed using the Pearson χ test or the Fisher–Freeman–Halton test. The Friedman test was used to determine whether there were statistically significant differences in VAS scores between groups for the follow-up timepoints. If the statistical results of the Friedman test were significant, the Dunn–Bonferroni multiple comparison test was used to establish the follow-up period causing the difference. Unless otherwise stated, results with *p* < 0.05 were statistically significant. However, the Bonferroni correction was used to control for type I error in all possible multiple comparisons.


**Informed consent:** All patients were informed and enrolled after obtaining written consent.
**Ethics approval:** The study was approved by the T.C. Ministry of Health Ankara Bilkent City Hospital 2nd Ethics Committee, numbered E2-21-228 (clinical trial approval no. NCT05152602, start date 01/10/2022, and last update posted on 01/10/2023).

## 3 Results

There was no significant difference between the groups in terms of demographics, including mean age, female to male distribution, BMI averages, concomitant disease, and median operation time (*p* > 0.05; Table 1).

**Table 1:** Socio-demographic and clinical characteristics

	Control group (*n* = 17)	Right ESP block group (*n* = 20)	Bilateral ESP block group (*n* = 17)	*p*-value
**Age (years) (mean ± SD)**	48.70 ± 12.02	47.90 ± 13.98	46.41 ± 12.64	0.872*
**BMI (kg/m** ^**2**^ **) (mean ± SD)**	27.97 ± 5.71	27.39 ± 5.64	29.00 ± 5.21	0.677*
**Operation duration (min) (mean ± SD)**	82.41 ± 19.43	77.30 ± 25.56	72.70 ± 18.57	0.432*
**Gender (** ***n*** **, %)**				0.732**
Female	8 (47.1)	12 (60.0)	9 (52.9)	
Male	9 (52.9)	8 (40.0)	8 (47.1)	
**ASA (** ***n*** **, %)**				0.104**
1	5 (29.4)	3 (15.0)	8 (47.1)	
2	12(70.6)	17 (85.0)	9 (52.9)	
**Additional disease (** ***n*** **, %)**				0.286**
No	9 (52.9)	9 (45.0)	12 (70.6)	
Yes	8 (47.1)	11 (55.0)	5 (29.4)	
Total	71 (100.0)	67 (100.0)	66 (100.0)	

*One-way ANOVA test.

**Chi-square test.

**Figure 1:**
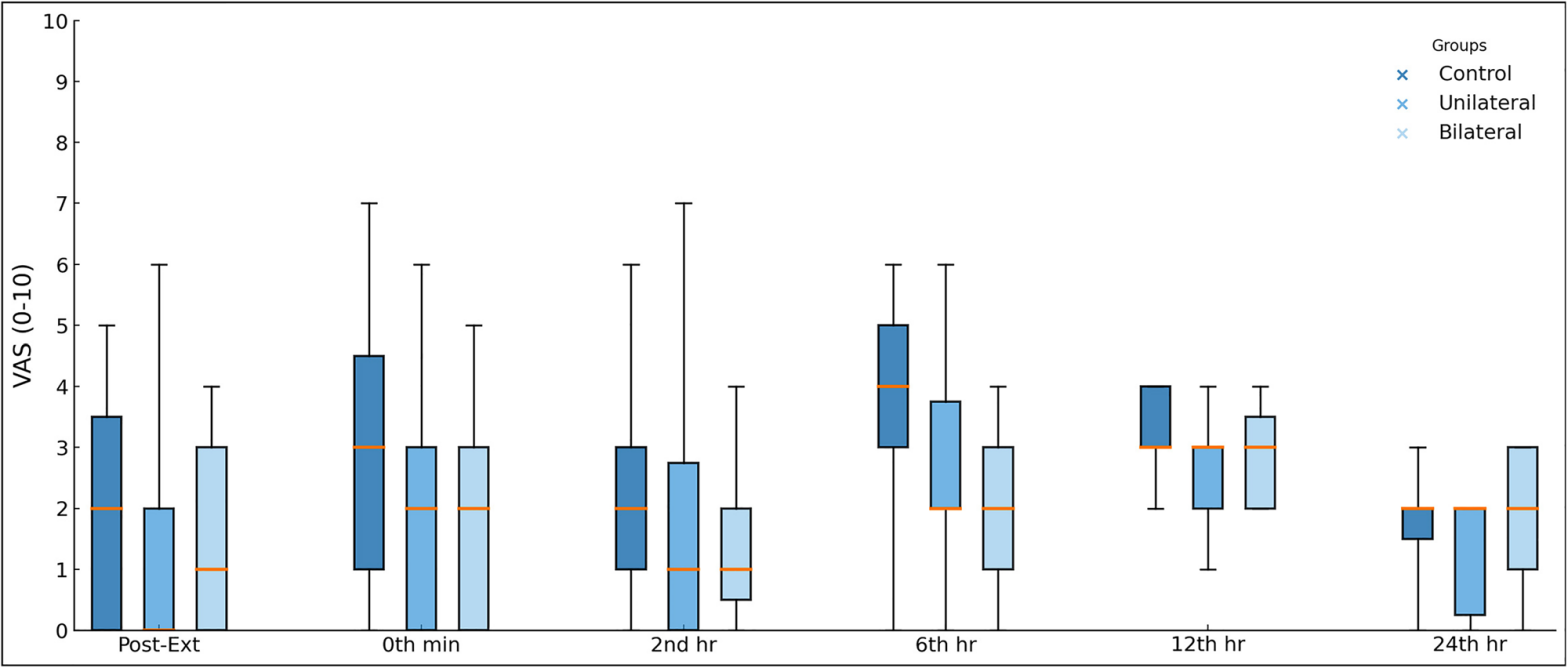
Box-plot graph for VAS scores in terms of study groups according to the follow-up times. The horizontal orange lines in the middle of each box indicate the median, while the top and bottom borders of the box mark the 25th and 75th percentiles, respectively. The whiskers above and below the box mark the maximum and minimum values for VAS scores.

At 6 h post-surgery, the VAS scores of the bilateral ESPB group were significantly lower than the control group (*p* < 0.001; Table 2). There was no significant difference between the control and unilateral ESPB groups (*p* = 0.019) and unilateral ESPB and bilateral ESPB groups (*p* = 0.836) in terms of VAS levels at 6 h post-surgery (Figure 1). There was no significant difference between the groups in terms of VAS values at 24 h post-surgery (*p* = 0.096; Table 2).

**Table 2:** VAS scores according to groups and follow-up times

	Control (*n* = 17)	Unilateral (*n* = 20)	Bilateral (*n* = 17)	*p*-value^†^
Post-extubation	2 (0–3.5)^a^	0 (0–2)^e^	1 (0–3)	0.412
Post-op 0 min	3 (1–4.5)	2 (0–3)	2 (0–3)	0.267
Post-op 2 h	2 (1–3)^b^	1 (0–2.75)	1 (0.5–2)^f^	0.156
Post-op 6 h	4 (3–5)^A,a,b,c^	2 (2–3.75)	2 (1–3)^A^	**<0.001**
Post-op 12 h	3 (3–4)^d^	3 (2–3)^d,e^	3 (2–3.5)^f^	0.043
Post-op 24 h	2 (1.5–2)^c,d^	2 (0.25–2)^d^	2 (1–3)	0.101
*p*-value^‡^	**<0.001**	**<0.001**	**<0.001**	

Data; displayed as median (25th percentile–75th percentile). Bold indicates the difference between the control group and the bilateral ESPB group was statistically significant (*p* < 0.001).

^†^Comparisons between groups at each follow-up time, according to Kruskal–Wallis test, Bonferroni correction, results for *p* < 0.0071 were considered statistically significant.

^‡^Comparisons between the follow-up times within the groups, Friedman test, Bonferroni correction for *p* < 0.0167 were considered statistically significant.

^A^The difference between the control group and the bilateral ESPB group was statistically significant (*p* < 0.001).

^a^Statistically significant difference between post-extubation and post-op 6th hour (*p* < 0.001).

^b^Post-op 2nd hour, the difference between the post-op 6th hour and the 6th hour was statistically significant (*p* = 0.007).

^c^Difference between post-op 6th hour and post-op 24th hour statistically significant (*p* < 0.001).

^d^The difference between post-op 12 h and post-op 24 h was statistically significant (*p* < 0.0167).

^e^The difference between post-extubation and post-op 12th hour was statistically significant (*p* = 0.005).

^f^Post-op 2nd hour to post-op 12th hour, the difference between the two is statistically significant (*p* < 0.001).

**Figure 2:**
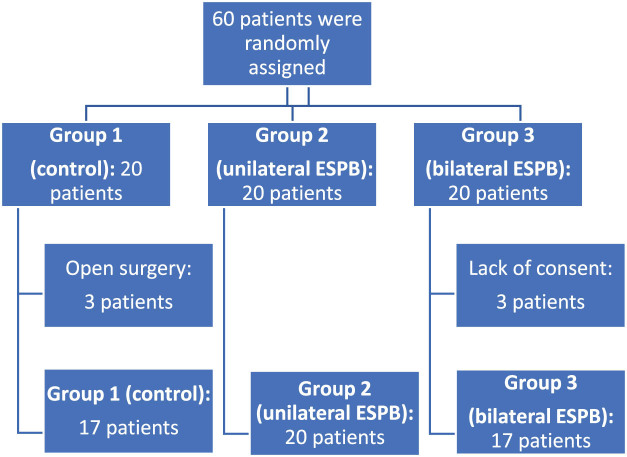
Flow chart of the study.

There was no significant difference between the groups regarding additional analgesic requirement, incidence of nausea and vomiting, shoulder pain, or discharge time in days (*p* > 0.05; Table 3). Tramadol use in the bilateral ESPB group was lower than in the control group (*p* = 0.003). Although the 24 h total tramadol use was lower in the unilateral group compared with the control group; however, the difference was not significant (*p* = 0.081; Table 3). In addition, there was no significant difference between the unilateral ESPB and bilateral ESPB groups (*p* = 0.924).

**Table 3:** Groups in terms of tramadol consumption amount in the first 24 h, shoulder pain, nausea, vomiting, and duration of discharge

	Control (*n* = 17)	Unilateral (*n* = 20)	Bilateral (*n* = 17)	*p*-value
Additional analgesic requirement	8 (47.1%)	10 (50%)	7 (35.0%)	0.531^†^
Discharge time (days) (*n*, %)				
1	10 (58.8)	12 (60.0)	9 (52.9)	0.634**
2	6 (35.3)	8 (40.0)	8 (47.1)	
3	1 (5.9)	0 (0.0)	0 (0.0)	
Nausea and vomiting	7 (41.2%)	3 (15.0%)	4 (23.5%)	0.197^¶^
Shoulder pain	0 (0.0%)	2 (10.0%)	0 (0.0%)	0.323^¶^
Total amount of 24 h tramadol	300 (200–300)^a^	200 (100–200)	100 (50–272.5)^a^	**0.003** ^‡^

Bold indicates the difference between the control group and the bilateral group was statistically significant (*p* = 0.003).

^†^Pearson’s *χ* ^2^ test.

^‡^Kruskal–Wallis test.

^¶^Fisher–Freeman–Halton test.

**Chi-square test.

^a^The difference between the control group and the bilateral group was statistically significant (*p* = 0.003).

## 4 Discussion

In this study, bilateral ESPB provided significant advantages over the control group and had lower pain scores and less tramadol use. Although unilateral ESPB also provided some advantages over the control group, no significant difference was observed. VAS scores were lower in the bilateral ESPB group compared with the control group at 6 h post-surgery. The recruitment of additional analgesics in the bilateral ESPB group was lower than the control group.

Reducing the side effects of postoperative analgesia has been a key goal in pain management. Opioids cause nausea, vomiting, and delayed recovery and discharge [7]. Multimodal analgesics with peripheral blocks reduce opioid use and side effects, and also reduce opioid requirements [4]. Nevertheless, paracetamol and non-steroidal anti-inflammatory drugs are the preferred pharmacological agents for laparoscopic cholecystectomy as stated in the recently updated PROSPECT guidelines. It was recommended that transversus abdominis, subcostal blocks, and peritoneal local anesthetic infiltrations are added when basic analgesia was not an option. It is possible that, despite its absence from the current discussion, the ESPB will eventually become an essential element in the management of somatic and visceral pain [8]. In a meta-analysis by Park et al., which included 6 randomized controlled trials and 314 patients, subcostal transversus abdominis plane (TAP) block and wound infiltration with local anesthetics were compared in terms of postoperative analgesia. More effective analgesia was provided in the patient group who underwent subcostal TAP block compared with the wound local anesthetic infiltration group [9].

The analgesic mechanism of ESPB is not fully understood but might involve paravertebral, epidural, or intercostal spread of local anesthetic. It is suggested that local anesthetic could spread to the paravertebral, epidural, or intercostal spaces, potentially providing both somatic and visceral analgesia, which might explain the analgesic effects observed in our study.

Ivanusic et al. suggest that when a local anesthetic is injected into the erector spinae muscle, the muscle undergoes craniocaudal elongation. Furthermore, the local anesthetic penetrates the costotransverse foramen and exerts its effects on both the dorsal and ventral branches via paravertebral and epidural spread [10]. Schwartzman et al. established the contralateral distribution of local anesthetic by examining the circumferential spread of local anesthetic into the epidural space with unilateral ESPB using magnetic resonance imaging [11]. Tulgar et al. [12] reported the unexpected spread of bilateral sensation as detected by an examiner masked to the test results, followed by a contralateral anesthesia using a local anesthetic, which clarified the results by examining the epidural space and including the dorsal rami via an alternative, posterior vertebral structure route. A previous investigation suggested that adequate analgesia could be achieved with unilateral ESPB [13].

Similar to our findings, Cesur et al. [14] showed that a bilateral ESPB is more efficacious than a unilateral ESPB in patients undergoing elective laparoscopic cholecystectomy. Additionally, the bilateral ESPB reportedly results in a reduction in the amount of opioids used and a decrease in the incidence of postoperative shoulder pain; however, this study did not identify any significant difference in the incidence of shoulder pain.

Similarly, a meta-analysis indicates that bilateral ultrasound-guided ESPB might be a suitable option for reducing the use of opioids and the time to the first administration of analgesia in adults undergoing laparoscopic cholecystectomy [15]. Vrsajkov et al. reported that the bilateral ESP block has been shown to provide superior postoperative analgesia and a reduction in the requirement for opioids following laparoscopic cholecystectomy [16].

These results were associated with better local anesthetic distribution and higher total amount of local analgesic in the bilateral ESPB group compared with unilateral ESPB. Our findings are in line with previous reports of the analgesic benefits of ESPB in abdominal surgeries. However, conflicting results in the literature underline the need for larger, multicenter trials. Clinicians should consider both the anatomical characteristics of the patient and the expertise of the practitioner when selecting ESPB as a component of multimodal analgesia. Future research should explore different volumes, concentrations, and techniques to optimize the block. On the one hand, the optimal concentration and type of local anesthetic are also not well established, although the doses of these blockers effectively influence the distribution of sensory blockers. Tulgar et al. [12] were performed with 0.25% bupivacaine 20cc. Aksu and Gürkan [17] injected 0.5 mL/kg 0.25% bupivacaine into the erector spinae plane (maximum dose was 4 mL per side). Gürkan et al. [18] administered 30 mL of 0.25% bupivacaine for breast surgery unilaterally at the T4 level. Altıparmak et al. [19] injected 20 mL of 0.25% bupivacaine as ESPB bilaterally at the T7 level. Unilateral injections of 30–40 mL of 0.25% bupivacaine have been observed to produce bilateral ESPB.

Enhanced recovery programs that aim to reduce opioid requirements and potential opioid-related side effects during the perioperative period are very important. The ESPB, which is part of a multimodal analgesia approach, is reportedly more effective than standard analgesia protocols in patients undergoing laparoscopic cholecystectomy and could, therefore, improve the quality of perioperative analgesia [20].

One of the limitations of this study was the lack of consideration of PCA morphine dose, which could be considered a deficiency. Also, there was a lack of data regarding postoperative PCA morphine use. Such data would have allowed a more comprehensive assessment of analgesic efficacy. Future studies should incorporate PCA morphine use as an outcome measure to better evaluate the opioid-sparing effects of ESPB. However, the adequacy of tramadol as an analgesic in laparoscopic cholecystectomies is acknowledged in the literature. The most important limitation of this study was the limited number of patients. Studies with larger samples are needed. Another important limitation was the absence of dermatome mapping to assess the extent of sensory blockade. Dermatome evaluation would provide valuable insight into the distribution and effectiveness of the ESPB. We recommend including objective sensory assessments in future studies to better understand the mechanism of action. While the mechanism of action of ESPB remains unclear and there is a possibility that even systemic absorption of the local anesthetic could have an effect, more detailed studies are required. Our study focused on the first 24 h of the postoperative period. As such, long-term outcomes related to pain control and functional recovery were not evaluated. Future studies should include long-term follow-up to assess the sustained effects of ESPB on postoperative recovery. Patients with BMI greater than 35 were excluded to maintain procedural consistency and reduce technical variability. However, this exclusion limits the generalizability of our findings. Future studies should consider broader inclusion criteria or subgroup analyses to assess the block’s efficacy in different patient populations.

The findings in this study are in line with previous reports demonstrating the analgesic benefits of ESPB in abdominal surgeries. However, conflicting results in the literature underline the need for larger, multicenter trials. Clinicians should consider both the anatomical characteristics of the patient and the expertise of the practitioner when selecting ESPB as a component of multimodal analgesia. Future research should explore different volumes, concentrations, and techniques to optimize the block.

## 5 Conclusions

There is currently no consensus on several key aspects of ESPBs, such as the optimal dose, infusion method, ideal local anesthetic concentration, and the required volume for single-shot or catheter applications. One of the main challenges remains the difficulty in predicting the distribution of local anesthetics. Despite these uncertainties, evidence suggests that a bilateral ESPB could be more effective than a unilateral ESPB for acute pain management. Although no statistically significant difference was observed in VAS scores between the two techniques, the bilateral approach could still offer clinical advantages, particularly in reducing postoperative opioid consumption. Therefore, bilateral ESPB should be considered a valuable component of multimodal analgesia strategies.
